# Compound-Specific δ^15^N Amino Acid Measurements in Littoral Mussels in the California Upwelling Ecosystem: A New Approach to Generating Baseline δ^15^N Isoscapes for Coastal Ecosystems

**DOI:** 10.1371/journal.pone.0098087

**Published:** 2014-06-02

**Authors:** Natasha L. Vokhshoori, Matthew D. McCarthy

**Affiliations:** Ocean Sciences Department, University of California Santa Cruz, Santa Cruz, California, United States of America; CSIR- National institute of oceanography, India

## Abstract

We explored δ^15^N compound-specific amino acid isotope data (CSI-AA) in filter-feeding intertidal mussels (*Mytilus californianus*) as a new approach to construct integrated isoscapes of coastal primary production. We examined spatial δ^15^N gradients in the California Upwelling Ecosystem (CUE), determining bulk δ^15^N values of mussel tissue from 28 sites between Port Orford, Oregon and La Jolla, California, and applying CSI-AA at selected sites to decouple trophic effects from isotopic values at the base of the food web. Bulk δ^15^N values showed a strong linear trend with latitude, increasing from North to South (from ∼7‰ to ∼12‰, R^2^ = 0.759). In contrast, CSI-AA trophic position estimates showed no correlation with latitude. The δ^15^N trend is therefore most consistent with a baseline δ^15^N gradient, likely due to the mixing of two source waters: low δ^15^N nitrate from the southward flowing surface California Current, and the northward transport of the California Undercurrent (CUC), with^15^N-enriched nitrate. This interpretation is strongly supported by a similar linear gradient in δ^15^N values of phenylalanine (δ^15^N_Phe_), the best AA proxy for baseline δ^15^N values. We hypothesize δ^15^N_Phe_ values in intertidal mussels can approximate annual integrated δ^15^N values of coastal phytoplankton primary production. We therefore used δ^15^N_Phe_ values to generate the first compound-specific nitrogen isoscape for the coastal Northeast Pacific, which indicates a remarkably linear gradient in coastal primary production δ^15^N values. We propose that δ^15^N_Phe_ isoscapes derived from filter feeders can directly characterize baseline δ^15^N values across major biochemical provinces, with potential applications for understanding migratory and feeding patterns of top predators, monitoring effects of climate change, and study of paleo- archives.

## Introduction

Isotope spatial gradients, or *Isoscapes*, are maps of systematic isotope variation and provide important biogeochemical information. Isoscapes are becoming increasingly important tools to characterize major biogeochemical zones and gradients in the ocean, and have been also used in ecological studies to help constrain animal migration and fish stock patterns (e.g., [1], [2]). Isoscapes of nitrogen (N) stable isotope values (δ^15^N) can be particularly informative, because such measurements have the potential to identify major ocean transitions between eutrophic/mesotrophic and oligotrophic regions, the balance of fundamental N cycle processes (e.g., N fixation vs. denitrification), and also basic ecological and food web relationships across major habitat zones. For example, water-column denitrification has a very large isotope effect (*ε*) of 25–30‰ [3] which greatly increases the δ^15^N value of all organisms in areas where this process is important. However, this could be rapidly changing in many ocean regions, linked to oceanographic climate events associated with a shifting climate (e.g., [4] Detailed isoscapes can ultimately provide a link between biogeochemical process and larger food webs, a key for understanding marine ecosystems. This is especially critical at a time when both natural and anthropogenic perturbations may be rapidly shifting fundamental biogeochemical processes (e.g., [5]), and potentially entire food web structures [6], [7].

However, the information potential inherent in δ^15^N values also presents significant challenges for interpretation of bulk δ^15^N values of organic matter. First, isoscapes are typically constructed from measurements in secondary or higher consumers. This approach provides a temporally integrated measurement; however, by definition, it also results in measured δ^15^N values being offset from “baseline” δ^15^Nvalues of primary production, since the ^15^N content of a consumer increases substantially with each trophic transfer. An average trophic enrichment factor (TEF) of ∼3.4‰ is often assumed [8]–[10], however it has been shown that the TEF values in fact vary substantially: not only between species, but also depending on tissue type, life stage, growth rate, and a host of other factors [10]–[12]. Further, for many oceanographic applications, such as understanding shifting gradients in primary production or N cycle processes, it is really the “baseline” δ^15^N value that is of primary interest (i.e., the δ^15^N value of primary production or N sources at the base of food webs). Because the bulk isotope value in a consumer is the combined signal of the baseline value *and* subsequent trophic effects, it is extremely difficult to isolate either factor with confidence.

Compound-specific isotope analysis of amino acids (CSI-AA) is a rapidly evolving technique that can address many inherent issues with bulk isotope data. For δ^15^N values, a seminal study by McClelland & Montoya (2002) demonstrated strong differential ^15^N enrichment of different groups of amino acids (AA) with trophic transfer. One group of AA has strongly elevated δ^15^N values with each trophic transfer (∼4–8‰), and are now termed the “Trophic AAs.” A second group of AA, now termed the “Source AAs,” in contrast has relatively constant δ^15^N values with trophic transfer, and so largely preserves δ^15^N values from the base of the food web. This pattern of AA differential enrichment has now been verified across a wide range of photoautotrophs and primary consumers [13], [14] and also in higher trophic organisms [15]–[19].

Most δ^15^N CSI-AA studies to date have focused on nitrogen isotopic values of two main AAs: phenylalanine (Phe), as the best indicator of baseline δ^15^N value, and glutamic acid (Glu), as the best indicator for relative trophic transfer. The relative predictability of ^15^N offsets between Glu and Phe with trophic transfer has also led to an explicit equation now used widely to calculate CSI-AA based trophic position (*see methods*). Based on these findings, CSI-AA patterns (δ^ 15^N_AA_) have now been used to not only estimate trophic position (TP) [16], [20], [21] and to trace source or microbial re-working of organic N sources [4], [19], [22]–[24], and animal movement across broad ocean basins [19], [25]. Taken together, CSI-AA work to date strongly suggests that if δ^15^N_AA_ is applied in appropriate heterotrophic organisms, the source AA should be able to indicate baseline δ^15^N isoscapes, decoupled from influence of trophic transfer.

The California mussel (*Mytilus californianus*) is a sessile resident of intertidal zones, which continuously filters particulate organic matter (POM). As such, mussels correspond closely to an ideal “baseline indicator” organism (i.e., a long-lived primary consumer; [10]). Because mussels temporally integrate filtered POM into their tissues and shells over annual to decadal timescales, they have been widely used as both sentinel organisms for marine pollutants (e.g., Mussel Watch Project: http://ccma.nos.noaa.gov/about/coast/nsandt/musselwatch.aspx), as well as to attempt reconstruction of ocean water composition and conditions [6]–[10], [26]–[29]. In contrast, many other organism types have important drawbacks for constructing representative isoscapes. For example, highly mobile top predators may rapidly transit distinct biogeochemical zones (e.g. [8]–[15], [18], [19], [30]), and thus attenuate isotopic variability. In contrast, short-lived organisms (such as zooplankton) can be assumed to not move widely, but because of relatively fast growth rates and rapid N turnover times may not integrate variation, but rather are subject to short temporal isotopic changes in the environment (e.g. [16]). Because of their sessile nature, cosmopolitan distribution, and continuous integration of water column food sources, mussels have major advantages as a potential basis for coastal isoscapes.

Here we examined δ^15^N_AA_ patterns in California mussels (*Mytilus californianus*) across 10 degrees of latitude in the coastal zone of the California Upwelling Ecosystem (CUE). The CUE is part of the greater California Current System (CCS), and is a highly dynamic region where we would anticipate not only large potential variation in baseline δ^15^N values, but also the potential for rapid future change linked to a warming climate [26], [28], [29]. Our overall goal was to explore whether δ^15^N values of source AA, and in particular Phe (δ^15^N_Phe_), may can serve as a new, direct proxy for constructing isoscapes of integrated δ^15^N values of primary production within highly dynamic coastal regions. We compared bulk δ^15^N and δ^15^N_ AA_ patterns in mussels to first test dependence of bulk isotopic variability on baseline δ^15^N values, using CSI-AA to constrain variations in TP. We also compared mussel δ^15^N values with literature values in more offshore sample types (zooplankton and POM), to examine if our results may also apply to the larger CCS. Our results indicate that source AA values in mussels are likely represent a direct record of variation in baseline δ^15^N values, and suggest that in the CA coast region isoscapes based on δ^15^N_Phe_ closely follow variations in nitrate δ^15^N values.

## Methods

### Sample Collection and Preparation

#### Ethics Statement

California mussels (*Mytilus californianus*) analyzed for this study were collected from 28 different sites between Coos Bay, Oregon and San Diego, California (Table 1), under a permit provided by the California Department of Fish and Wildlife.

**Table 1:** Collection sites and bulk δ^15^N values for *Mytilus californianus.*

Site	Identifier	Habitat Type	Latitude	Longitude	*n*	*δ* ^15^N	SD
Humbug Mtn./Port Orford, OR	HMPO	Rocky	42°43'N	124°28'W	6	7.8	0.2
Meyer's Creek Beach, OR	MCPR	Rocky	42°18'N	124°25'W	5	7.4	0.3
Pelican State Beach, CA	PSB	Rocky	42°00'N	124°12'W	6	8.3	0.2
Lagoon Creek, CA	LC	Rocky	41°36'N	124°06'W	5	8.3	0.4
Humboldt Lagoon	HL	Rocky	41°13'N	124°06'W	5	8.6	0.2
Luffenholtz Beach, CA	LB	Rocky	41°02'N	124°07'W	6	8.2	0.3
Point Cabrillo Lighthouse, CA	PCL	Rocky	39°21'N	123°48W	5	8.4	0.3
Schooner Gulch	SG	Rocky	38°52'N	123°39'W	5	9.2	0.4
Stillwater Cove Marine	SWC	Rocky	38°32'N	123°17'W	5	9.8	0.2
Bodega Bay, CA	BB	Rocky	38°19'N	123°04'W	5	11.1	0.5
Pacifica, CA	PAC	Rocky	37°39'N	122°29'W	5	10.0	0.1
Half Moon Bay, CA	HMB	Jetty	37°29N	122°27'W	5	9.3	0.3
Davenport, CA	DAV	Rocky	37°00'N	122°10'W	5	10.0	0.3
Santa Cruz, CA	SC	Rocky	36°56'N	122°03'W	6	10.9	0.2
Moss Landing, CA	ML	Jetty	36°48'N	121°46'W	4	10.2	0.2
Asilomar, CA	ASI	Rocky	36°38'N	121°56'W	5	9.4	0.3
Rocky Point, CA	RP	Rocky	36°24'N	121°54'W	6	10.0	0.4
Mill Creek, CA	MC	Rocky	35°58'N	121°29'W	5	9.5	0.3
Morro Bay, CA	MB	Rocky	35°22'N	120°51'W	5	10.0	0.2
Gaviota, CA	GAV	Rocky	34°28'N	120°13'W	4	10.4	0.1
Santa Barbara, CA	SB	Rocky	34°23'N	119°42'W	5	11.4	0.2
Ventura, CA	VEN	Jetty	34°16'N	119°17'W	3	10.7	0.1
Malibu, CA	MAL	Rocky	34°01'N	118°45'W	6	10.1	0.2
Topanga, CA	TOP	Rocky	34°02'N	118°34'W	4	10.7	0.4
Venice Beach, CA	VB	Jetty	33°58'N	118°28'W	4	11.3	0.4
San Clemente, CA	SCL	Pier	33°25'N	117°37'W	5	10.3	0.3
Oceanside, CA	OCE	Jetty	33°13'N	117°23'W	5	10.8	0.3
La Jolla, CA	LAJ	Rocky	32°50N	117°16'W	5	11.5	0.3

Reported δ^15^N values represent averages for all individuals collected from each location (4 to 6 individuals, 35–45 cm size range; *see methods*). “Identifier” indicates the abbreviation used in text for specific sites; “habitat type” indicates if site was natural rocks or an artificial structure; n =  number of individuals collected; SD is the standard deviation for the δ^15^N values measured from all individual mussels sampled from a given site.

**Figure 1:**
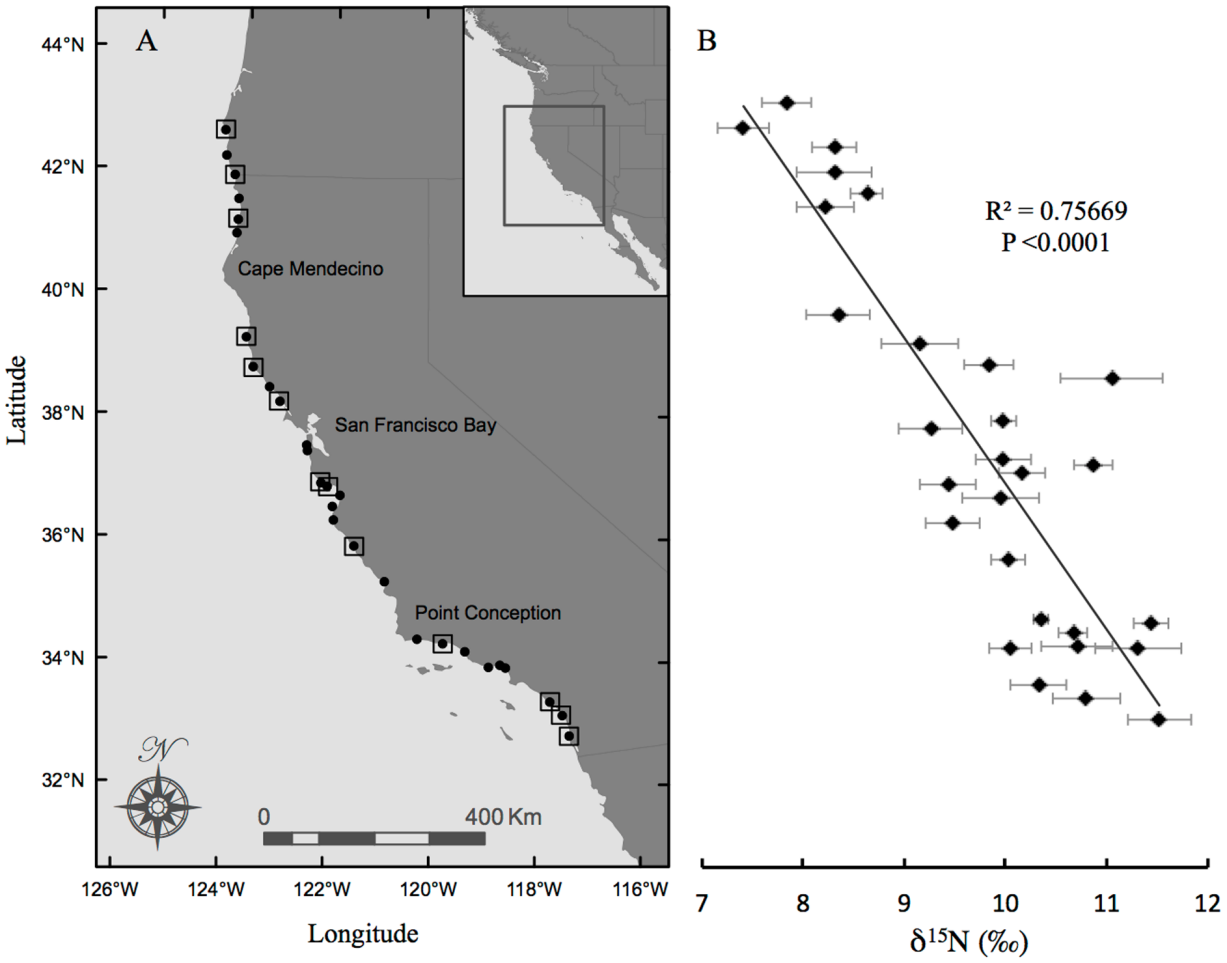
Collection sites and bulk δ^15^N values. Site-specific bulk δ^15^N values of *Mytilus californianus* as a function of latitude, in context of a map of collection sites on the California coast. (A) Filled circles indicate all sampling sites, and map locations correspond directly to bulk analysis values in panel B; open squares represent sites chosen for compound-specific isotope analysis. (B) Filled diamonds indicate average δ^15^N for five individuals sampled from each site; error bars indicate ±1SD. Regression line and statistics indicate strong linear relationship of δ^15^N values versus latitude.

Mussels were collected in the winter (Dec – Feb) of 2009–2010. Sites were chosen to be approximately evenly distributed along the CA coastline, with ∼80 km geographic separation between each sampling site. Our main goal here was to sample mussels from a wide geographic range across the CCS, although for observing finer scale local or regional variations, a finer-scale sampling strategy would like be required. Typically 5 individual mussels were collected from each site, all between 30–40 mm maximum shell length, which were immediately placed on dry ice until further preparation. The adductor muscle of each individual was dissected for analysis. This tissue was selected because isotopic values in muscle tissue have shown relatively long turnover times; based on past growth data, mussels of this size would be expected to integrate approximately annual variability in suspended food source isotopic values for each location sampled [31]. The dissected adductor tissue was carefully separated from other tissue types, rinsed with deionized water, refrozen, and then freeze-dried for 48 hrs. Lipids were removed following the methods of Dobush et al. (1985) [32], using petroleum ether in a Dionex Accelerated Solvent Extractor (Bannockburn, IL). Finally, in preparation for CSI-AA, composite samples were made from a subset of 13 collection sites (Fig.1a). For each location chosen for CSI-AA (based on the bulk δ^15^N record), 1±0.05 mg of lyophilized tissue was weighed and combined for each individual mussel (*n* = 5). Further CSI-AA preparation proceeded as described below.

### Bulk Stable Nitrogen Analysis

Stable nitrogen isotope analyses were conducted using standard protocols in the Stable Isotope Lab at the University of California, Santa Cruz (UCSC-SIL). Briefly, homogenized muscle tissue of each individual was weighed into tin capsules and combusted. Isotope values determined on a Carlo Erba 1108 elemental analyzer (Lakewood, NJ) coupled to a Thermo Finnigan Delta Plus XP isotope ratio mass spectrometer (San Jose, CA) (EA-IRMS). Analytical error associated with this measurement was typically <±0.15‰ based on sample replicates. Stable isotopes are reported using standard delta (δ) notation in parts per thousand (‰):δ^15^N =  [(R_sample_/R_standard_) − 1] ×1,000, where R is the ratio of heavy to light isotope, R_sample_ is from the sample, and the R_standard_ is atmospheric N_2_ (air) for carbon, as provided by pulses of calibrated CO_2_ reference gas. For details on correction calculations and normalization to international standards refer to UCSC-SIL website (http://es.ucsc.edu/~silab/index.php).

### Compound-specific amino acid δ^15^N analysis

Amino acid δ^15^N values were measured as Trifluoroacetyl isopropyl ester (TFA-IP) AA derivatives, following protocols described in detail elsewhere (e.g.,. Briefly, samples were hydrolyzed (6 N HCl, 20 hr at 110°C) under nitrogen, and TFA derivatives subsequently prepared from free AA using a modified version of the protocol described by Silfer (Silfer et al. 1991): isopropyl esters were made with a 1∶5 mixture of Acetyl Chloride (AcCl):2-propanol (110°C, 60 minutes), and then acylated using a 1∶3 mixture of Dichloromethane:Trifluroacetyl acetate (DCM:TFAA) (100°C, 15 minutes). Derivatized AAs were dissolved in DCM to a final ratio of approximately 4 mg of original tissue to 250 µl DCM.

After derivatization, samples were analyzed by a Varian gas chromatograph coupled to a Finnegan Delta-Plus isotope ratio mass spectrometer (GC-IRMS). AAs were separated using a 50 m, 0.32 ID Hewlett Packard Ultra-1 column with 1 µm film thickness. Under our analytical conditions, δ^15^N values could be reproducibly measured for alanine (Ala), aspartic acid + asparagine (Asp), glutamic acid + glutamine (Glu), leucine (Leu), isoleucine (Ile), proline (Pro), valine (Val), glycine (Gly), lysine (Lys), serine (Ser), phenylalanine (Phe), threonine (Thr), and tyrosine (Tyr) (Fig. S4). Most AAs were measured with a standard error of <1.0‰ (based on *n* = 4 injections), and the average mean deviations for individual AA δ^15^N measurements across all tissue sample replicates was 0.5‰.

### Amino Acid Categories and Trophic Position Calculations

In all results and discussion, measured AA are grouped into one of three categories: “Trophic” vs. “Source” (*after* Popp et al. 2007 [15]) and Thr alone is designated as a “metabolic” AA (*after* Germain et al. 2013 [18]). The measured Trophic AA (with large expected enrichment in ^15^N with trophic transfer) were Glu, Asp, Ala, Ile, Leu, Pro and Val. The measured Source AA (with expected little to no change in δ^15^N at higher trophic levels) were Phe, Gly, Ser, Lys and Tyr. The δ^15^N values of Thr exhibit an apparent inverses isotopic fractionation with trophic transfer, however are also highly variable with organism type [18], so this AA is considered outside the basic Trophic vs. Source division.

Based on this framework, we used unweighted averages of AA groupings, as well as specific TP estimates, to analyze our data. Average Trophic and Source δ^15^N values were calculated:
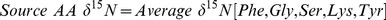
(1)

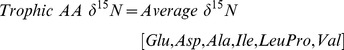
(2)


For explicit TP calculations, we used the “canonical” AA's (Glu and Phe) to calculate TP of mussels in the CUE, after Chikraishi et al. 2009:
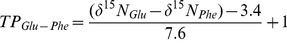
(3)where, δ^15^N_Glu_– δ^15^N_Phe_ are measured values, +3.4 is the assumed isotopic difference between the Glu and Phe in primary producers (also referred to as the β value), and +7.6 is the assumed ^15^N enrichment in Glu relative to Phe with each trophic transfer from food source to consumer, also called the Δ value [13].

### Statistical analyses and calculations

Statistical analyses (e.g., Hierarchical cluster analysis and Analysis of Covariance) were conducted using the JMP statistical software package (SAS Inc., Version 10). We used Arc-GIS Spatial Analyst (version 10.1) to produce visual isoscapes of the CUE. Our first model is based on the line for δ^15^N_Phe_ values vs. latitude (y = −0.3328x+20.053, R^2^ = 0.63592). Our second model (Fig. S1) is based on one-dimension of δ^15^N values along the latitudinal extent of sampling area and interpolates between data points of known δ^15^N values and to 100km offshore.

## Results

### Bulk δ^15^N values

Bulk δ^15^N values in the adductor muscle of *Mytilus californianus* ranged from 7.4‰ to 11.5‰ (Table 1). Bulk δ^15^N values were measured on tissue from multiple individual mussels (4–6, but typically 5) collected from each site to gauge intra-site variability in individuals. Standard deviations on average δ^15^N values for individual mussels from the same sites ranged from 0.1 to 0.5‰ (Table 1). The average standard deviation for all intra-site comparisons, across all locations, was 0.3‰. This value is close to EA instrument error (∼0.2 ‰), and therefore indicates an extremely small degree of variation in individual mussel δ^15^N values within specific sites, implying instead strong homogeneity of δ^15^N values for mussel populations. When plotted as a function of latitude, the average bulk δ^15^N values for mussels from each site have a strong linear trend (Fig. 1B). The average bulk δ^15^N values increase by 0.41‰ per degree of latitude from north to south (R^2^ = 0.755 and *P*<0.0001). We note that all sampling sites were located in exposed waters, and variable habitat type (i.e. rocky, jetty, etc.; see table 1) did not appear to be a major factor in the overall latitudinal trend in isotopic value.

### Amino Acid δ^15^N values

Of the 28 sites measured for bulk analysis, 13 were chosen for CSI-AA (Fig 1A). Samples for CSI-AA were chosen first to obtain relatively even geographic spacing, with specific locations within geographic regions then selected to capture maximum offsets in the north to south bulk δ^15^N trend (Fig. 1B). Measured δ^15^N values for individual AAs ranged from −1.0‰ to 16.0‰ (Table 2). In all samples, Thr was distinct, with the lowest δ^15^N values, the Source AA group always had intermediate values, and the Trophic AAs always had the highest δ^15^N values (Table 2; Fig. S2). Over the entire data set, the range of the averaged Source AA δ^15^N (*see methods*) was 5.1 to 10.3‰ (SD = 1.3‰, *n* = 69) and the averaged Trophic AA values are 10.3 to 15.9‰ (SD = 0.4‰, *n* = 91). While precision for individual AA δ^15^N measurements varied (Table 2), it was typically <1‰, with the average analytical standard error across all AA we measured at all sites as 0.8‰ (*n* = 160).

**Table 2:** Compound specific amino acid δ^15^N values for *Mytilus californianus*.

							Trophic	Source												Metabolic
Site	*n*	Bulk *δ* ^15^N	Average Trophic	Average Source	Trophic Position	Glu -Phe	Glu	±	Asp	±	Ala	±	Ile	±	Leu	±	Val	±	Pro	±	Gly	±	Ser	±	Lys	±	Tyr	±	Phe	±	Thr	±
HMPO	4	7.8	10.3	5.1	1.3	5.9	12.1	1.3	10.2	0.3	9.8	0.3	9.4	0.6	11.6	0.2	7.6	0.8	11.4	0.4	2.9	0.7	6.0	0.7	nd	nd	nd	nd	6.3	1.4	−2.2	0.3
PSB	4	8.3	11.8	6.3	1.3	5.8	12.0	0.7	10.1	0.4	13.1	0.1	11.7	0.4	12.7	0.2	9.4	0.8	13.2	0.3	6.0	1.0	6.7	1.0	nd	nd	nd	nd	6.2	0.4	−2.1	0.8
HL	4	8.6	12.3	7.6	1.3	5.6	12.7	0.1	10.5	0.2	12.4	0.4	12.3	0.5	13.5	0.2	10.3	1.0	14.0	0.2	7.1	1.2	7.0	1.2	11.0	0.7	6.0	0.5	7.2	0.6	−0.7	0.2
PCL	4	8.4	13.4	5.4	1.6	8.1	14.3	0.1	11.6	0.3	14.4	0.2	13.2	0.7	14.7	0.3	11.0	1.0	14.6	0.5	5.6	0.6	5.6	0.6	3.9	2.2	5.5	0.5	6.2	0.7	−3.1	0.3
SG	4	9.2	12.5	5.8	1.2	5.0	10.5	0.6	11.7	0.2	14.4	0.3	12.3	0.9	13.4	0.1	13.2	0.9	11.6	0.5	6.8	0.1	7.6	0.1	nd	nd	3.2	1.0	5.5	0.3	−0.1	0.3
BB	4	11.1	15.9	8.2	1.8	9.5	17.4	0.8	14.3	0.4	18.1	0.5	16.0	0.5	16.7	0.4	16.5	0.6	12.4	0.5	9.3	1.0	8.6	1.0	8.6	0.6	6.6	0.8	7.9	0.4	−0.9	0.6
DAV	4	10.0	14.0	7.5	1.6	8.0	16.2	0.3	14.0	0.2	12.8	0.6	12.0	0.9	16.4	0.5	10.8	0.9	15.6	0.3	7.4	0.6	9.6	0.6	6.7	0.9	5.8	1.9	8.2	0.8	−0.5	0.5
SC	4	10.9	12.3	5.1	1.7	8.4	15.3	1.1	13.5	0.1	12.1	0.5	12.0	0.7	14.7	0.3	6.0	1.3	12.6	0.5	5.3	1.0	8.4	1.0	nd	nd	nd	nd	6.9	1.0	0.4	0.3
MC	4	10.0	13.3	8.0	1.3	5.4	13.7	0.2	11.8	0.2	13.5	0.7	13.4	0.8	14.4	0.2	11.3	0.2	14.6	0.2	8.3	0.3	7.4	0.3	nd	nd	nd	nd	8.3	0.5	−1.1	0.2
SB	4	11.4	15.1	9.0	1.6	8.3	16.3	0.1	14.0	0.1	16.5	0.2	14.6	0.2	15.7	0.1	15.5	0.6	13.2	0.3	10.0	0.3	9.9	0.3	9.8	0.2	7.4	0.7	8.0	0.4	1.5	0.3
SCL	4	10.3	12.7	8.4	1.2	4.7	13.4	0.1	12.1	0.1	12.9	0.1	11.9	0.1	12.9	0.1	12.7	1.0	13.1	0.1	9.2	0.3	9.2	0.3	9.0	0.6	5.7	1.2	8.7	0.4	2.6	0.2
OCE	4	10.8	13.2	10.3	1.0	3.3	13.9	0.2	12.3	0.1	13.3	0.2	13.0	0.4	13.7	0.2	12.4	0.2	13.8	0.2	11.1	0.2	9.8	0.2	12.1	0.8	7.6	0.8	10.7	0.4	3.2	0.1
LAJ	4	11.5	14.3	*7.7*	1.4	6.6	15.4	0.3	13.4	0.2	15.1	0.1	13.8	0.2	15.2	0.2	14.6	0.8	12.4	0.5	8.8	0.8	9.8	0.2	4.9	1.4	6.3	1.6	8.8	0.7	−0.4	0.3

δ^15^N_ AA_ for individual amino acids from 13 individual collection sites, ± the analytical standard deviation from replicate injections (*see methods*). Site abbreviations, bulk δ^15^N values, and “n” refer to data for specific sites selected for CSI-AA (Table 1). Trophic, Source and metabolic categories, amino acid abbreviations, and calculated averages for Trophic and Source AA's and Trophic Position are as defined in text

**Figure 2:**
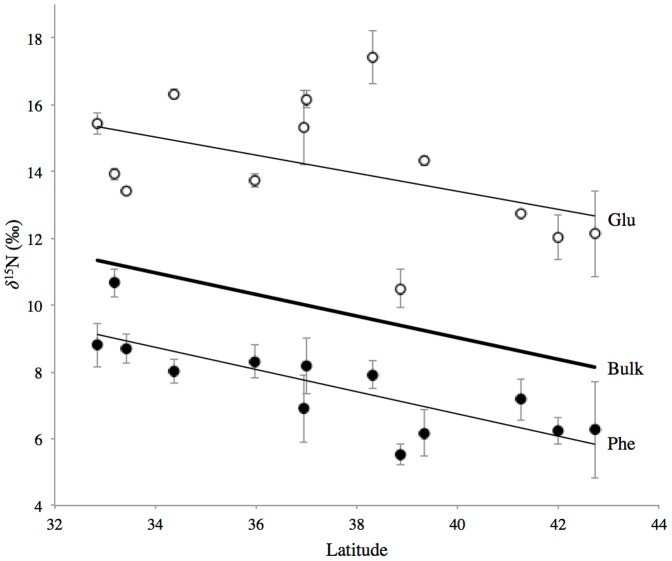
Latitudinal trends in Glu and Phe δ^15^N values compared with bulk δ^15^N. Bold solid line indicates regression for bulk δ^15^N values for all sites sampled (as in Fig. 1), thin solid lines indicate linear regressions for Glu (open circles) and Phe (filled circles) (δ^15^N_Glu_ = −0.270x+24.211, δ^15^N_Bulk_ = −0.313x+21.527, and δ^15^N_Phe_ = −0.329x+19.927). ANCOVA analysis indicates that all three share a common slope within error (effects test, *P*<0.0050). Error bars for Glu and Phe indicate analytical standard deviation for CSI-AA performed on a composite sample for all individual mussels from each site (*see methods*).

We focused on Glu and Phe δ^15^N values as the best proxies for Trophic and Source AA groups, respectively, as has been indicated by a number of recent papers [19], [33]–[36]. Glu and Phe δ^15^N values both correlated significantly with average values for Trophic and Source AA groups respectively (Phe vs. average Source AA, R^2^ = 0.782; *P* = 0.0006; Glu versus average Trophic AA's, R^2^ = 0.546, *P* = 0.0049), confirming the validity of this approach (see also [25]). Both Phe and Glu δ^15^N values also tracked changes in bulk δ^15^N with latitude (Fig. 2). The δ^15^N values of Phe and bulk adductor muscle had a strong and significant linear relationship with latitude (*P* = 0.0028 and *P* = 0.0011, respectively). In contrast, there was more variability in the Glu data. The relationship of Glu vs. bulk δ^15^N was not significant at 95% confidence (*P*>0.05), however a Fit Model run of Analysis of Covariance shows that δ^15^N values of bulk, Phe and Glu all share a common slope (effects test, *P* = 0.0050); in other words, the slope of δ^15^N change with latitude for Glu and Phe are not significantly different from the slope of bulk δ^15^N change with latitude.

### Mussel Trophic Position and Trophic Enrichment Factors

The TP of mussels calculated using Eq. 3 ranged from 1.0 to 1.8 with an average TP of 1.4±0.3 (Table 2). TP had no correlation with latitude (*P* = 0.706), indicating that despite local variability, mussels' suspended POM food sources had similar average TP in all CA coastal regions. Across all mussel samples analyzed with CSI-AA, the average δ^15^N_Glu_ – δ^15^N_Phe_ offset was 6.5‰. Prior work indicates that these mussels feed primarily on microalgae [37]–[39], coupled with data for δ^15^N_Glu_ – δ^15^N_Phe_ offsets in phytoplankton and marine macroalgae [13], [22], this average offset would indicate an average TEF_Glu-Phe_ for *Mytilus californianus* of 3.1‰ (Fig. S3).

## Discussion

This study investigated if δ^15^N CSI-AA values measured in mussel “bio-archives” can represent a new approach to understanding baseline δ^15^N patterns in dynamic coastal regions. We hypothesize that the potential for δ^15^N_AA_ values to decouple trophic shifts from baseline δ^15^N values may, for the first time, allow construction of isoscapes specifically for temporally integrated δ^15^N values for primary production, based tissue samples from heterotrophic organisms. Mussels were chosen for this study because they are sessile, filter-feeding organisms with tissue turnover rates integrating suspended POM food sources on monthly to annual timescales. Other studies have previously CSI-AA or bulk δ^15^N patterns in primary producers, zooplankton [31], or mobile top predators, to trace oceanographic processes [15]. However, as noted above, in many heterotrophic organisms multiple variables might complicate inferences about baseline isotopic signals. For plankton these include variability caused by shorter biochemical turnover rates, coupled with seasonal change in nutrient availability, light intensity and temperature fluctuate [16], while in higher trophic level animals factors such as migration or a mixed diet may dilute the desired signal in question [30]. Overall, we expect that sessile filter feeders such as mussels are likely to be are among the best organisms for baseline source records in systems where they occur.

### δ^15^N latitudinal gradient in the California Upwelling Ecosystem

We hypothesize that the strong δ^15^N gradients with latitude are driven by the mixing of two NO_3_^−^ source waters, coupled by upwelling in the CUE. In this region, northern low-^15^N water is brought south by surface flow of the main CCS [3], [40]. At the same time, southern source of elevated ^15^N water originating from the zone of denitrification in the ETNP [33], [34], [36], [41], [42] is brought north via the California Undercurrent (CUC). The source area for the CUC (approximately south of the tip of Baja CA peninsula) is one of the major persistent oxygen minimum zones (OMZ) in the world ocean [3], [40] accounting for 35–45% of global pelagic denitrification [36], [41]–[43]. Bacterial denitrification in the low-oxygen water columns has a very large positive fractionation factor (ε ∼20–30‰; e.g., [3], [34]), and therefore imprints a distinct signal in surrounding waters, such that subsurface NO_3_^−^ values for the southern CUC near the tip of Baja can approach +14‰ [36], [43]–[45]. The CUC then moves northward, with its flow attenuating as it progresses along the CA margin. The core of the CUC is near 150 m, directly in source-depth regions for upwelled waters [34], [43]. The main isotopic endmembers for inorganic nitrogen along our study region are therefore open Pacific nitrate from CCS (∼ 5‰, e.g. Sigman et al., 2009), mixed with the ^15^N enriched nitrate being carried northward by the CUC (∼9–10 ‰; [44]–[46]), and brought to the surface locally via upwelling. We note, however, that while all previous literature clearly indicates an expected change in baseline δ^15^N with latitude, it cannot indicate exact δ^15^N endmembers for the CA coast region we sampled. This is because of the relative paucity of direct nitrate ^15^N measurements, and the inherent temporal and geographical variation in these measurements, even within similar regions (e.g., [43], [46]–[48]).

A complimentary forcing for δ^15^N trends could therefore also be variability of upwelling intensity with latitude. The North American west coast is commonly described in terms of three distinct upwelling regions, characterized by differences in overall annual upwelling intensity: Baja California (21–30°N), continental US (30–48°N) and British Columbia and Alaska (48–60°N) [46], [47]. While winds generally increase northward, annual wind intensity is most consistent year-round south of Pt. Conception, strongest seasonally along central CA coast, and generally weakest north of Cape Blanco [39], [46]–[48]. Our study site does not cross all three of these main regions, however it seems possible that the transition between the southern and central zones of upwelling intensity could influence the overall latitudinal trend. Overall, while expected variation in average upwelling intensity is consistent with our observations of latitudinal δ^15^N change, wind forcing alone cannot not explain the clear linear decrease in δ^15^N values with increasing latitude.

Local denitrification is another process that could also contribute to regional δ^15^N baseline values. Water column denitrification in the CCS has been documented in borderland basins (such as in the Santa Barbara Basin, SBB; [47]), where water column exchange with the ocean is blocked by basin sills, causing basin water to become O_2_ deficient [34], [44], [47], [49]. It has also been shown to occur in areas along the Oregon coast, due to advection of oxygen-poor water masses onto continental shelves [50]. However, if local denitrification were a main factor driving relative δ^15^N values, we would predict far more localized δ^15^N variability. Therefore, while this cannot be ruled out as contributing to δ^15^N values for specific locations, it seems highly unlikely as the major forcing for such a regular gradient. Finally, both water temperature and sampling season might be considered as additional factors. As noted above, all mussels were collected in the winter season of 2009–2010. While it is possible that mussel metabolism may change throughout the year (high vs. low feeding seasons), the specific tissue analyzed (adductor muscle, *see methods*) and mussel size class were specifically selected to isotopically integrate over an approximate yearly time frame. This assumption is supported by preliminary data of samples collected in both summer and winter season of the same year for selected sampling sites, for which no significant effect on the observed latitudinal trend was observed [39], [45], [51]. Water temperature is also a general function of latitude in the CCS at all times of year. Change in water temperature might affect the isotopic gradient either directly via changing mussel metabolism, or indirectly as a proxy for upwelling strength. However, in contrast to the mussel N isoscape, the major temperature changes along the CA coast are not linear, but rather shift more strongly at the boundary of the Southern CA Bight, with temperatures generally much warmer south of Pt. Conception, and consistently much cold temperatures (due to stronger upwelling) in central and northern CA.

Overall, the strong latitudinal δ^15^N trend recorded in mussel tissues seems most consistent with the endmember mixing outlined above, consistent with both modeling and prior discrete sampling. For example, δ^15^N values of sediment traps and sediment cores contrasted between central CA vs. the Southern CA bight have indicated δ^15^N values are generally more enriched in the Southern CA bight vs. Northern CA, consistent with our measurements [34], [44], [49], [52], [53]. In addition, basin-scale modeling of δ^15^N variation [3], [45], [51], [54] also predict a south to north trend of decreasing baseline δ^15^N values, driven by the ETNP denitrification endmember. Our study therefore represents perhaps the strongest confirmation to date of both model predictions, and also prior discrete-location sampling results. However, no prior sample set has ever tested CCS latitudinal δ^15^N variation at such high resolution, based on an archive coupling unambiguous source location with approximately annual signal integration. In particular, the striking linear trend in our data is a novel, and also perhaps a surprising finding. This indicates that the diminution in ^15^N-enriched nitrate supply via the CUC (or relative mixing with southerly CCS) is remarkably regular in the CUE: across the 10 degrees of latitude that we sampled, δ^15^N change was remarkably consistent (0.41‰±0.04 per degree). We suggest that the ability to capture this regional trend at such high precision is linked to the integrative property of filter feeding consumers, as well as the longer-turnover tissue that we sampled. Overall, we propose that Fig. 1b indicates the integrated approximately annual gradient in ^15^N values in coastal CUE waters with latitude. If correct, this also suggests that surveys of costal mussel δ^15^N values might constitute a powerful new tool for constraining physical mixing and circulation models, since they would show the effective mixing of two source waters in great detail.

While satellite data has documented changing global ocean surface chlorophyll concentrations, leading to predictions of declining primary production in the world's oceans due to increased stratification associated with warming [49], [52], [53], [55], the effects of a warming climate on CCS biogeochemistry remain unclear. Some studies have proposed that some CCS zones are already showing an opposite trend of increasing productivity, linked to increased nutrient supply [3], [22], [28], [54]. If the ocean nitrate endmember were to increase over time, this should result in gradual decrease in δ^15^N values in the CUE, and potentially also a change in the slope of the clear latitudinal gradient we observe. At the same time, increasing stratification in CCS waters is also proposed as one main consequence of warming, and this has already been documented [13], [49], [56]. If this decreased the effective supply of CUC water and associated nitrate, it could also lead to lower δ^15^N values. However, the potential effects of natural climatic perturbations (e.g. El Nino Southern Oscillation – ENSO, and Pacific Decadal Oscillation – PDO cycles) are currently very difficult to decouple from longer term trends. Given that our current understanding of physical and biological responses of the CUE to a changing climate remains poor. Repeated sampling of mussels could provide a time and geographically integrated record of baseline isotopic change in this system, revealing longer term trends in the regional δ^15^N gradient, due to either natural fluctuations or climate change, at near annual resolution.

### Do coastal mussel δ^15^N data also reflect broader California Current δ^15^N values?

An important question is to what degree δ^15^N data derived from mussels may reflect isotopic values of broader coastal waters, as opposed to only littoral sources and process. This is likely to be a function of relative time scale: the time frame over which mussels integrate δ^15^N of primary production, vs. the mixing time scale for littoral water with more seaward coastal water masses. If water mixing is relatively rapid vs. sampled tissue isotopic turnover, then it is possible mussels would reflect isotopic values within the broader CUE, and possibly into shoreward extent of the CCS. In contrast, if upwelling and nutrient utilization in the littoral zones are rapid and strongly localized, then littoral mussel δ^15^N values could be mostly decoupled from values in more offshore coastal waters. To definitively address this question, an extensive sampling program would likely be required, comparing offshore/onshore POM isotope values with those recorded in mussel tissues.

**Figure 3:**
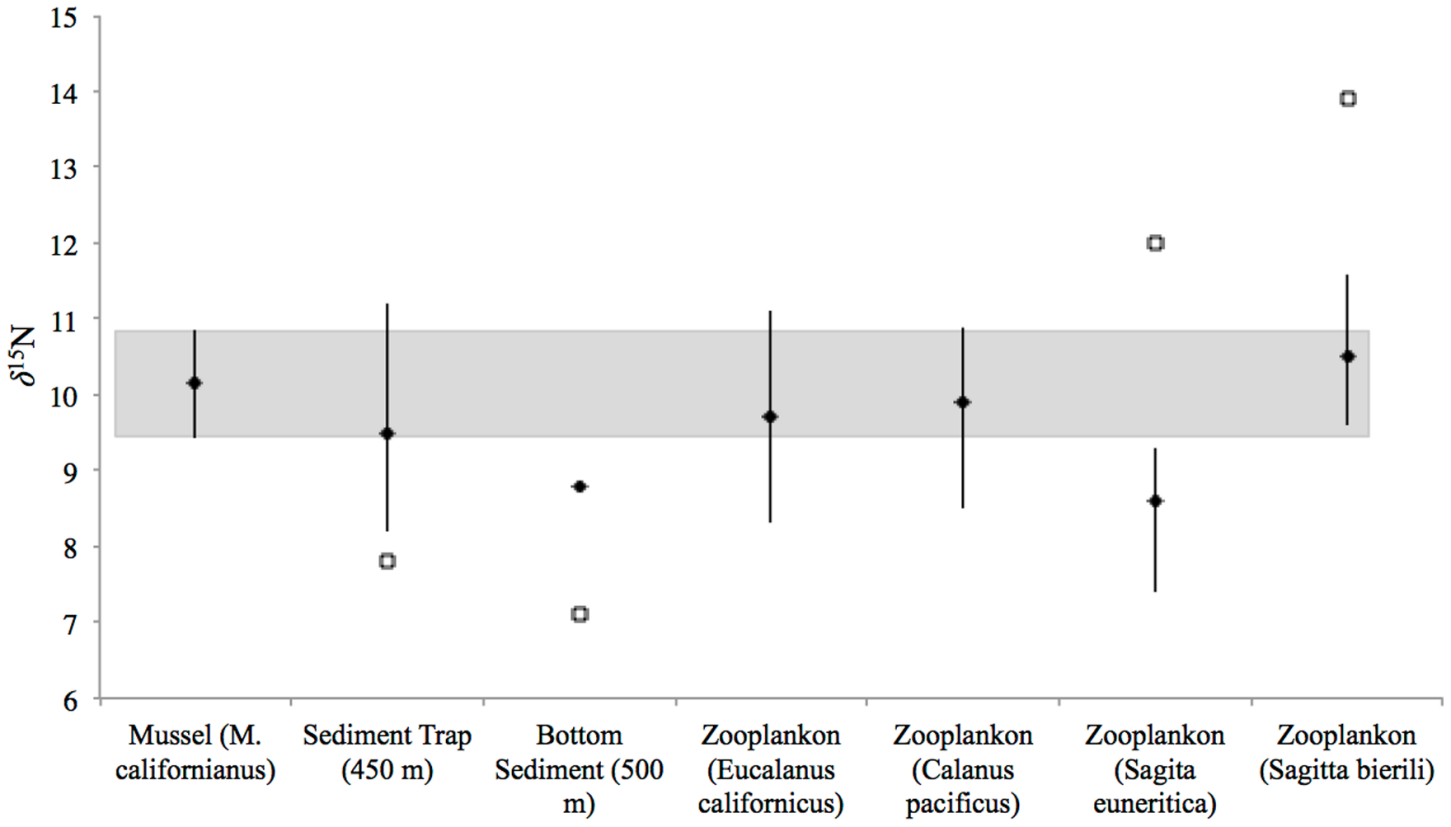
δ^15^N values for littoral mussels compared with offshore sample types in Monterey Bay region. δ^15^N values for *Mytilus californianus* (this study) compared with literature values for sediment trap and bottom sediments (Altabet et al. 1999), primary consumer zooplankton (*Eucalanus californicus* and *Calanus pacificus*) and secondary consumer zooplankton (*Sagitta euneritica* and *Sagitta bierii)* (Rau et al. 2003) from Monterey Bay. For literature sample types diamonds indicate values corrected for estimated trophic position, open squares are the reported average literature value. Shaded band indicates range of our measured δ^15^N values for mussels from three Monterey Bay sampling sites (SC, ML, ASI); for literature sample types error bars indicate main range for reported values.

However, for the well-studied Monterey Bay region, past work offers extensive data sets for both coastal and offshore δ^15^N values in both organisms and detrital OM. We therefore compiled δ^15^N values for a range of sample types from the Monterey Bay region, and compared these with δ^15^N values for mussels at our sampling sites in or near the bay (Fig. 3). Specifically, we compared average δ^15^N values for 2 herbivorous and 2 carnivorous zooplankton species [22], [45], [57]and also OM in sediment traps (450 m) and surface sediment (950 m) samples[13], [15], [16], [24], [34], [35], [39]. Because many of these samples are not primary consumers, δ^15^N change due to trophic transfer must be taken into account for any direct comparison. We therefore assumed that mussels and herbivorous zooplankton, as primary consumers would feed at the same TP as mussels (TP = 2), requiring no correction. For carnivorous zooplankton (secondary consumers) we assumed one additional trophic transfer (TP = 3), and therefore adjusted reported δ^15^N values by 3.4‰ (the most broadly accepted average bulk TEF value). For sediment trap and surface sediment samples, we used recent results from Monterey Bay long-term sediment trap records, which have indicated an average trophic position (determined by CSI-AA) of 1.6 (Sherwood and McCarthy, *unpublished data*; also similar to TP for POM at Station ALOHA,[20], [33], [45]). We therefore adjusted both trap and surface sediment values for 1.5 TP (+1.7‰).

While we acknowledge that this approach can provide only a very general initial comparison, the results are nevertheless quite encouraging (Fig.3). For most sample types, the Monterey Bay adjusted values fall directly within the δ^15^N range for local mussels. This suggests that littoral mussels may in fact reflect δ^15^N values more broadly for local coastal waters. Given the high wind stress and mixing characteristic of the Central and Northern CA coasts, this may not be surprising. However, clearly this represents only a preliminary comparison, and rests on a range of assumptions that remain to be fully tested (e.g. that time of sampling is relatively unimportant, or that bulk TEF values are accurately estimated). To fully explore the potential of littoral mussels as integrators of coastal isoscapes, we suggest a synoptic sampling program comparing offshore/onshore mussel isotopic values will be required.

### Mussel trophic position

**Figure 4:**
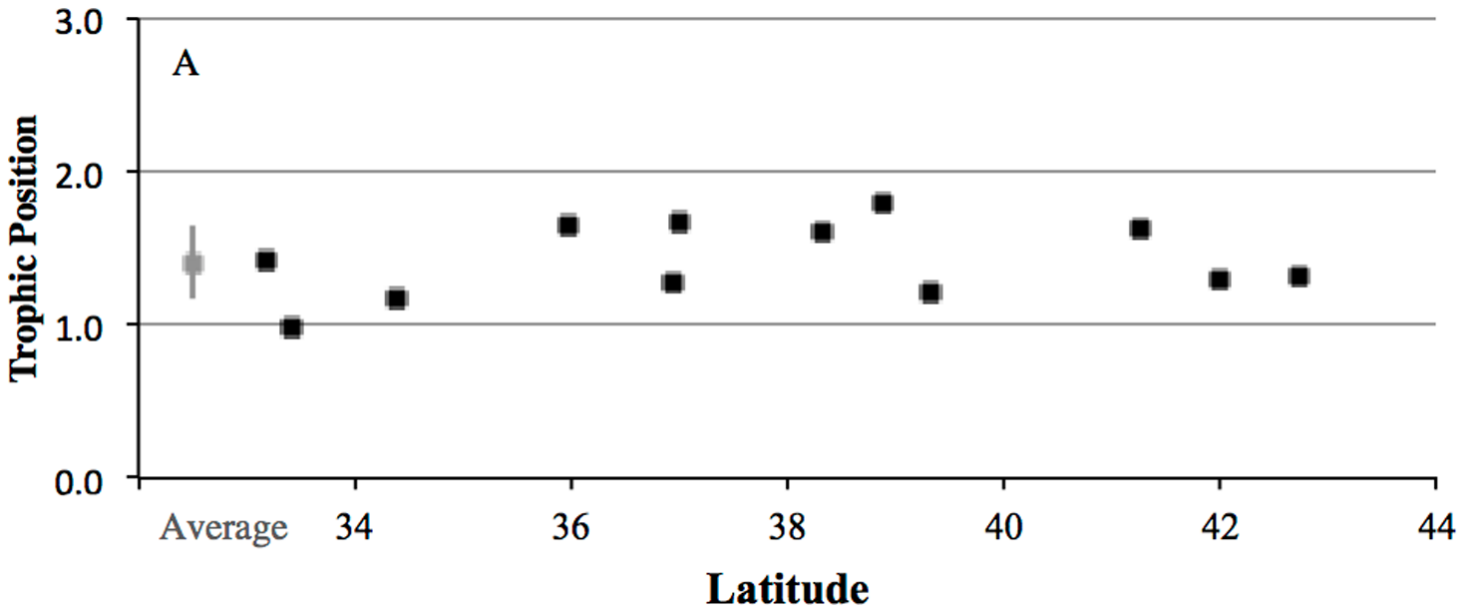
CSI-AA based trophic position for mussels from California Coast. Calculated trophic position (TP) for composites of mussels from the sites selected for CSI-AA (filled squares) and average TP ± 1SD for all mussels sampled along CA coast (grey square)

CSI-AA provides a unique opportunity to decouple the effects of trophic transfer from δ^15^N values at the base of the food web. Based on differential enrichment behavior of the Trophic vs. Source AAs introduced above [13], [15], [16], [21], [24], [35], [39], [52], CSI-AA allows for a direct assessment of the role for TP variation may play in bulk δ^15^N value trends. Our calculations of mussel TP for an extended population along the entire CA coast represents, to our knowledge, the first wide-ranging CSI-AA survey of any filter feeding mollusk population in nature. The lack of any trend in TP with latitude (Fig. 4) indicates that mussels spanning the entire CA coast feed at a very similar TP, therefore likely on similar food sources, independent of location. The consistency of TP is interesting, given the previously documented localized variation in δ^13^C values for mussels from these same locations (Vokhshoori et al. in press). The similarity of TP from all locations therefore supports the conclusion that δ^13^C variation is primarily driven by changes in baseline δ^13^C values. Since TP does not change with latitude also strongly supports our basic hypothesis that the overall δ^15^N trend with latitude (Fig. 1b) is also driven by north to south variation in baseline δ^15^N values, most likely linked to nitrate δ^15^N values.

However, the exact TP values (average TP = 1.4±0.2; Fig. 4a) calculated using the standard Glu-Phe approach (*see methods*, Eq. 1), also are lower than would be expected. Mussels predominately feed on POM derived from primary production [58], [59], so as primary consumers the mussel TP values should be at least 2. There are at least two possible explanations for the lower TP indicated by CSI-AA. One relates to non-algal food sources that might contribute to mussel AA, for example detrital POM (Vokhshoori et al. in press) or non-algal primary production sources. For example, some primary producers can have different baseline CSI-AA patterns from marine microalgae (e.g., seagrasses; [2], [13], [15], [37], [39], such that substantial contributions from non-microalgal sources would change the calculated TP calculated using the standard equation (*see Text S1*). However, δ^13^C_AA_ source fingerprinting applied to these same mussels has verified a dominantly marine microalgal diet [38], [39], in agreement with ecological expectations diet (e.g.,[3], [33]). This suggests that if different source δ^13^C_AA_ patterns do account for the offset, it is more likely due to variations among different microalgal groups. Given the relatively limited current δ^13^C_AA_ data on different marine algal lineages, this is possible. An alternate possibly, is that the change in Glu δ^15^N values with trophic transfer in mussels may be smaller than the TEF factor now most commonly applied (*and assumed in* *Equation 1* *; see methods*). Accumulating evidence now suggests that CSI-AA trophic enrichment factors may be specific for different groups of organisms [18], [34], [36], [40]–[42], [60], however TEF values offsets have so far been documented only higher TP predators. Ultimately, distinguishing between these possibilities is beyond scope of our current data, however we provide a broader explanation of the underlying issues in Text S1.

Overall, however, it is important to stress that apparently low TP values for mussels do not bear in any significant way on our main observations. Specifically, the constant TP with latitude indicated by CSI-AA data is independent of exact TP estimates. However, TP data does suggest that controlled feeding experiments with filter-feeding mollusks, together with a more extensive survey of variation in the δ^15^N-AA in different algal types, will be needed to clearly interpret TP values derived from mussel tissue or shells. Such work might also be important for future potential development of CSI-AA patterns in archeological mussel shells as potential paleoceanographic bioarchives.

### AA-CSIA: new tool to reconstruct primary production δ^15^N isoscapes

As noted above, all literature to date has indicated that Phe δ^15^N values are closely linked to δ^15^N values at the base of the food web, such that δ^15^N_Phe_ values in a heterotroph can be used to estimate δ^15^N values of average primary production sources [3], [5], [15], [16], [34]–[36], [43], [44]. Given that these mussels feed almost uniquely on microalgae [37]–[39], [46]–[48], [59] we therefore hypothesize that mussel δ^15^N_Phe_ should represent a temporally integrated value for δ^15^N of coastal phytoplankton production. Sampling δ^15^N_Phe_ in mussel populations along a coastline should therefore yield, for the first time, a way to construct an integrated isoscape of baseline δ^15^N values.

**Figure 5:**
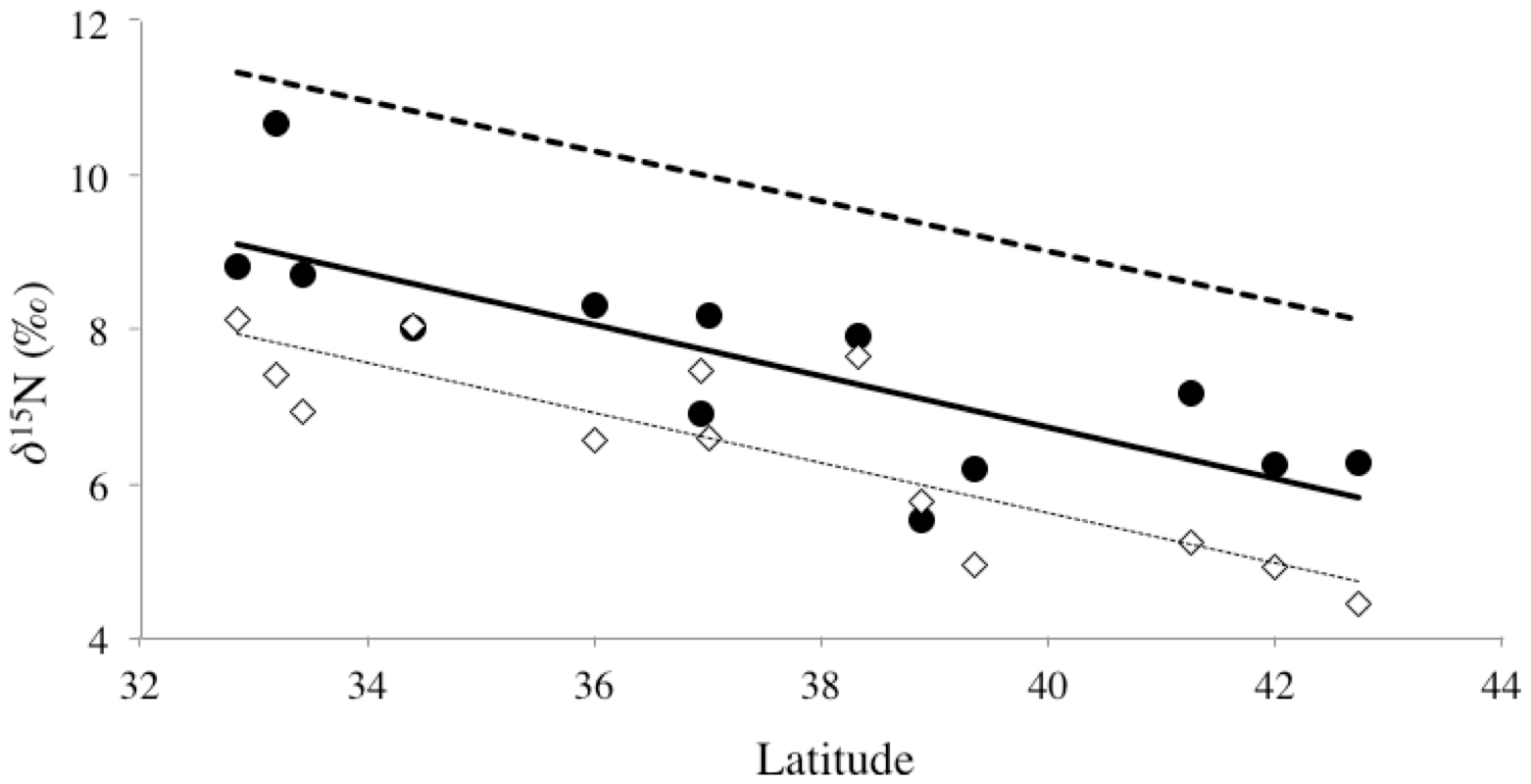
Two approaches for the estimation of baseline CUE δ^15^N values from mussel isotopic data. The CSI-AA approach, based on average values for δ^15^N_Phe_ (filled circles, solid regression line) predicts average baseline δ^15^N values most consistent with expected NO_3_^−^ δ^15^N gradients along the CA coast. An alternate approach is based on measured bulk δ^15^N values, adjusted for an assumed trophic position (open diamonds, dashed regression line). This approach cannot take into account either TEF or tissue-specific fractionations, and returns lower than expected values in most locations. The regression for measured bulk δ^15^N values in adductor muscle tissue (heavy dashed line) is provided for reference.

A direct comparison between bulk δ^15^N and δ^15^N_Phe_ values is one way to test this idea (Fig. 5). If we assume average complete NO_3_^−^ utilization for this region (at least over ∼annual time frames mussels integrate; e.g., [49], [52]), then the slope of the regression for bulk δ^15^N values (Fig. 1b) should also represent the *gradient* in NO_3_^−^ δ^15^N values along the CA coast. In this case, the “baseline” δ^15^N values should also be essentially equivalent with NO_3_^−^ δ^15^N values. However, the bulk δ^15^N relationship of course cannot directly represent baseline values, due trophic transfer enrichment factors, as well as tissue-specific offsets. In Fig. 5 we therefore compare two possible approaches for estimating the baseline δ^15^N values from measured tissue data. The first relies only on bulk δ^15^N results, and assumes a standard bulk TEF value of 3.4‰, for mussels feeding at TP = 2.0. This approach predicts baseline δ^15^N values are far more reasonable vs. the measured bulk tissue values (Fig. 5; open diamonds). However, the results also appear to underestimate δ^15^N of NO_3_^−^ along the CA coast (∼4.5 to 8‰ north to south), when compared with previous literature data [3], [22]. This may not be surprising, since by definition this approach cannot take into tissue-specific δ^15^N offsets, nor the actual TEF values for mussels. In contrast, our proposed CSI-AA approach uses δ^15^N_Phe_ values as a direct proxy baseline δ^15^N. The fact that the slope of δ^15^N_Phe_ vs. latitude is identical within error to bulk δ^15^N (*see results*) strongly supports the idea of a constant offset from primary production. The δ^15^N_Phe_ regression-derived baseline values are universally heavier than the bulk- δ^15^N derived data discussed above, and are a closer match for reported NO_3_^−^ values in northern vs. southern CA waters.

We therefore hypothesize that δ^15^N_Phe_ is ultimately more accurate representation of baseline δ^15^N, because it requires no assumptions about TEF values in any specific organism. However, in order to derive precise baseline δ^15^N predictions based on δ^15^N_Phe_, it will also be necessary to have robust calibrations for the offset between δ^15^N_Phe_ and average algal δ^15^N values. The close match we observe between the δ^15^N_Phe_ regression values and expected NO_3_^−^ δ^15^N values of this region (Fig.5; [13], [49]) strongly suggests this offset cannot be very large in this system. This would agree with recent analysis of δ^15^N_ AA_ patterns measured in a range of phytoplankton species [22], [45]. However, other work has indicated larger offsets in some macroalgal and also micro-algal species tested in feeding experiments [13], [39]. An alternate approach for the future also could be to derive more broad-based corrections, based on δ^15^N values of multiple Source AA. Together with indications regarding the importance of representative β values for TP calculations, this further underscores the need for future work aimed at a more robust understanding of δ^15^N_AA_ patterns across representative algal sources.

### Baseline δ^15^N isoscapes from CSI-AA data

**Figure 6:**
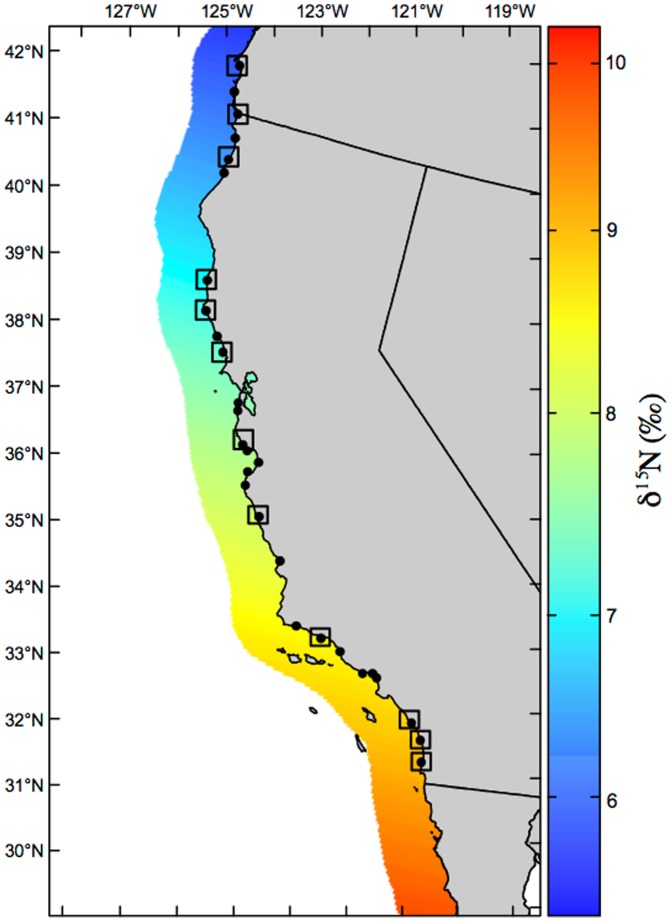
δ^15^N_Phe_ isoscape of the California Upwelling Ecosystem. Color gradient bar indicates δ^15^N values. Isoscape is based on the linear relationship of δ^15^N_Phe_ vs. latitude (R^2^ = 0.635, *P* = 0.0011). Squares represent sampling sites chosen for compound-specific isotope analysis; small black dots show all sampling sites for reference.

Taken together, these results suggest that CSI-AA has the potential, for the first time, to allow direct reconstruction of δ^15^N isoscapes of primary production, based on δ^15^N_Phe_ values measured in consumers. A baseline δ^15^N isoscape for the CUE (Fig. 6), derived on our δ^15^N_Phe_ values, represents to our knowledge the first such application. While the general *trend* of decreasing δ^15^N values with latitude is similar to broad trends predicted in regional or basin-scale models [45], we suggest that the new potential for CSI-AA to directly produce baseline δ^15^N isoscapes represents a major advance. Further, in initial data for selected resampling has shown that specific site-to-site offsets in bulk δ^15^N values have so far been highly reproducible [39]. This suggests that, while our initial CUE baseline isoscape is clearly based on relatively few locations, specific geographic variations are may also be meaningful (Fig. S1). While further sampling will be required to verify this conclusion, if mussel-derived δ^15^N values can indicate reproducible, fine scale geographic variation in baseline δ^15^N values, then our results suggest the potential to create highly detailed spatial maps of isotopic baselines, even in complex coastal environments.

Overall, a CSI-AA approach for constructing baseline isoscapes could have broad importance in both modern and paleoceanographic studies. While CSI-AA based isoscapes could also be generated from other consumers, we suggest that for coastal zones mussels may be a particularly useful bioarchive. The combination of ubiquitous occurrence in many coastal regions, relatively long-term integration of microalgal isotopic signatures, and unambiguous source locations together would provide strong confidence in geographic patterns. We suggest that the degree to which mussel-derived isoscapes also reflect more offshore coastal waters will be an important topic for future work. If our mussel data indicates broad similarity to near-shore coastal isotopic data as a general result (Fig. 3), then sampling within largely existing shore-based programs might rapidly produce detailed, annualized, baseline isoscapes for the entire CCS. Such data could be invaluable in understanding the changing environmental factors driving spatial variability within the CCS; for example the effects of ENSO and PDO cycle effects on baseline δ^15^N isoscape gradients, and also provide a more clear understanding of isotopic baselines needed to evaluate possible long-term trends linked to climate change. We also note that isoscapes constructed using CSI-AA from mussels also would not necessarily be limited to coastlines. Mussels frequently attach to the base of fixed moorings located offshore (e.g. http://www.mbari.org/oasis/index.html), and therefore might be used to examine temporal change in isoscapes in many offshore instrumented locations. Finally, our results also suggest potential for paleoceanographic reconstructions. Mussel shells are often the major species found in archeological middens widely distributed from Baja California to Alaska along the US west coast. If source AA δ^15^N patterns were also well preserved in archeological shell, this could potentially extend the reconstruction of coastal baseline isoscapes back through much of the Holocene.

## Supporting Information

Figure S1 **Alternate δ^15^N Isoscape approach.** Alternate δ^15^N Isocape of the California Upwelling Ecosystem, showing δ^15^N gradients between sampling stations. As in text Fig. 6 in main text, color gradient indicates δ^15^N values. However, this isoscape interpolates between δ^15^N at each specific site. While CSI-AA data coverage was not large in this study, preliminary re-sampling has indicated offsets are reproducible. While clearly additional sampling would be required to verify such variation, this approach directly suggests the potential for high resolution isoscapes that capture finer scale regional patterns. Given the ubiquity of mussels along the CA coast, as well as relative ease of sampling, such high resolution coastal isoscapes of baseline δ^15^N might be readily constructed.
(TIF)

Figure S2 **δ^15^N_AA_ patterns in the California Mussel (** ***Mytilus californianus*** **).** δ^15^N amino acid signatures of Mytilus californianus from 13 sampling sites selected for CSI-AA(values based on n = 4 analytical replicate injections). Absolute δ^15^N values normalized to the δ^15^N_Phe,_ so that patterns can be compared. Measured amino acids are categorized into Trophic, Source, and Metabolic (M), based on relative changes with trophic transfer (see main text). Site and amino acid abbreviations are as defined in main text. Overall δ^15^N_AA_ patterns conform closely to those expected from other heterotrophic organisms, with Trophic AA enriched in ^15^N vs. Source AA, and Thr strongly depleted in ^15^N.
(TIFF)

Figure S3 **Low CSI-AA based Mussel Trophic Position Results.** Relationships between measured δ^15^N_Glu-Phe_ values vs. expectations for standard CSI-AA TP equations. Measured δ^15^N_Glu-Phe_ of mussels are plotted vs. latitude (filled diamonds). Shaded bar on average value represents ± 1SD for entire data set. Assumed β values for primary producers are indicated by lower dotted line, (β_Glu-Phe_, 3.4 per mil). Commonly assumed TEF_Glu-Phe_ for a single trophic transfer for a primary consumer (7.6 per mil ) is represented by upper dashed line. Arrow represents Δ_Glu-Phe_ the theoretical isotopic enrichment from a TP1 to a TP2.
(TIFF)

Figure S4 **Representative chromatogram of a GC-IRMS analysis of amino acids.** Mussel amino acid gas chromatogram. A representative gas chromatogram of derivatized individual amino acids from *Mytilus californianus*. Abbreviations: Ala, alanine; Gly, Glycine; Thr, threonine; Ser, serine; Val, valine; Leu, leucine; Ile, isoleucine; Nor, Norleucine (internal standard); Pro, proline; Asp, aspartic acid, Met, Methionine; Glu, glutamic acid; Phe, phenylalanine; Lys, Lysine.
(TIFF)

Text S1 **Mussel trophic position discussion.**
(DOCX)
