# Supplementary figures and images for: Compound-Specific δ15N Amino Acid Measurements in Littoral Mussels in the California Upwelling Ecosystem: A New Approach to Generating Baseline δ15N Isoscapes for Coastal Ecosystems
Source: PLoS One. 2014 Jun 2;9(6):e98087. doi: 10.1371/journal.pone.0098087 (PMC4041574; doi:10.1371/journal.pone.0098087)

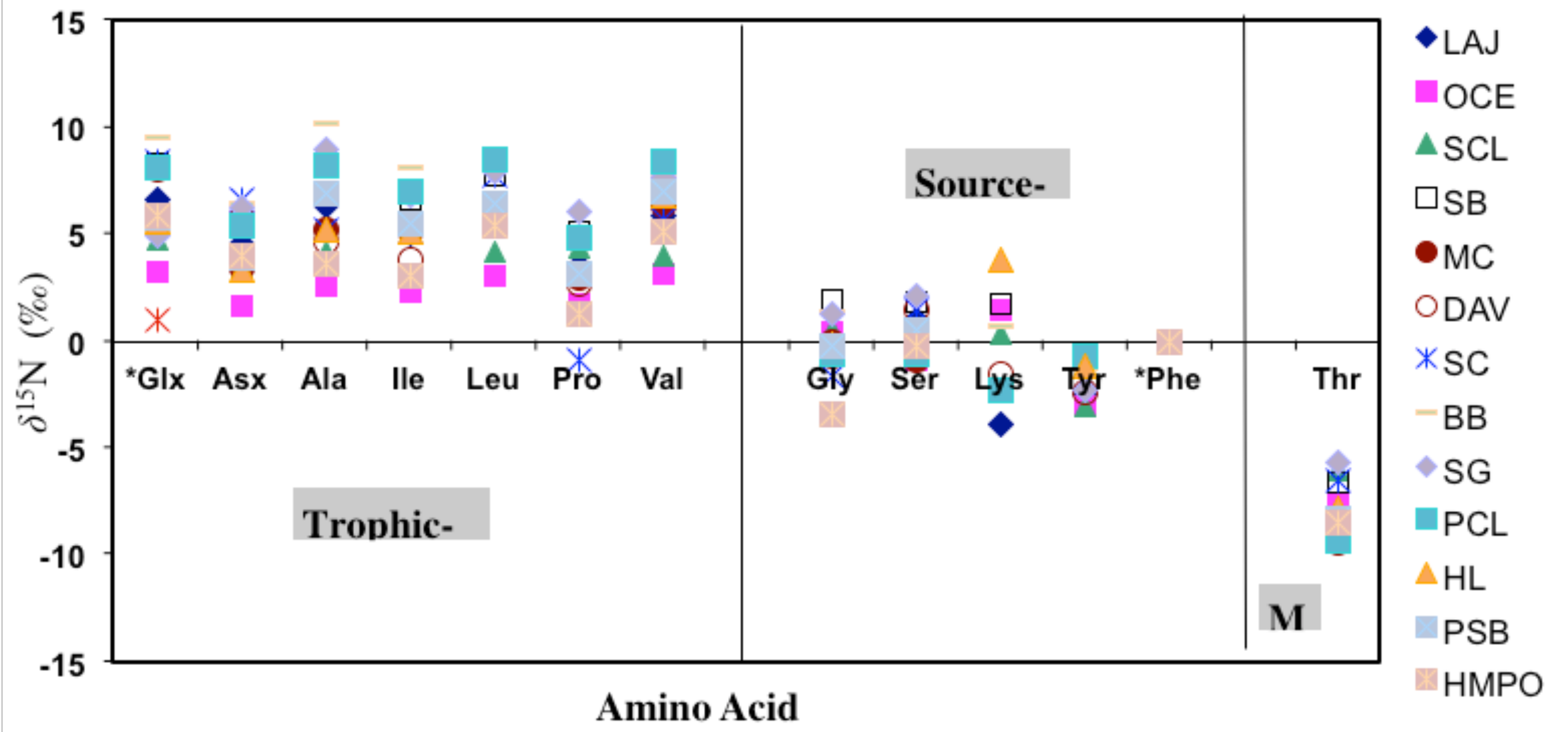

Supplement: Figure S1 — Alternate δ15N Isoscape approach. Alternate δ15N Isocape of the California Upwelling Ecosystem, showing δ15N gradients between sampling stations. As in text Fig. 6 in main text, color gradient indicates δ15N values. However, this isoscape interpolates between δ15N at each specific site. While CSI-AA data coverage was not large in this study, preliminary re-sampling has indicated offsets are reproducible. While clearly additional sampling would be required to verify such variation, this approach directly suggests the potential for high resolution isoscapes that capture finer scale regional patterns. Given the ubiquity of mussels along the CA coast, as well as relative ease of sampling, such high resolution coastal isoscapes of baseline δ15N might be readily constructed. (TIF) [file pone.0098087.s001.tif]

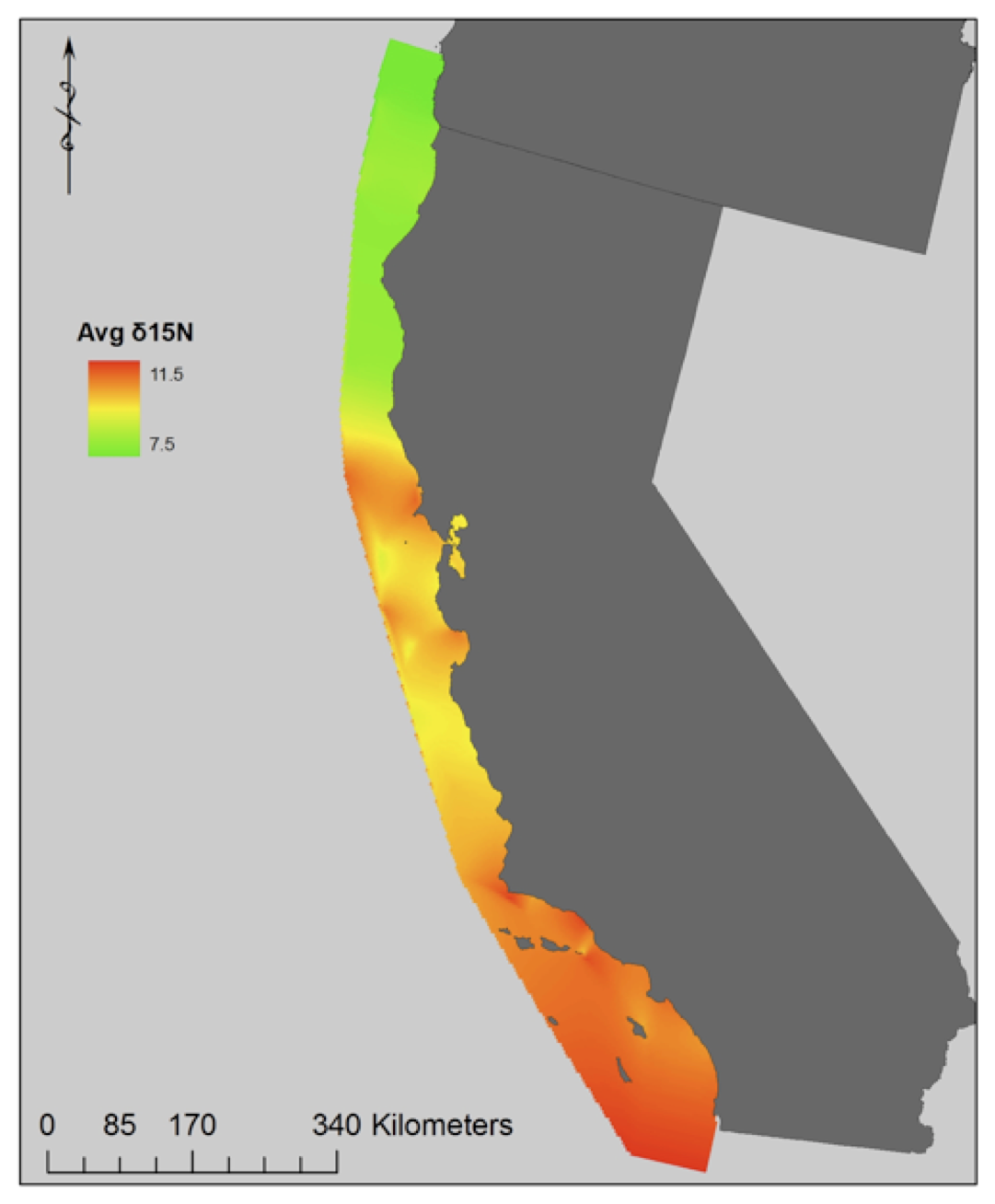

Supplement: Figure S2 — δ15NAA patterns in the California Mussel ( Mytilus californianus ). δ15N amino acid signatures of Mytilus californianus from 13 sampling sites selected for CSI-AA(values based on n = 4 analytical replicate injections). Absolute δ15N values normalized to the δ15NPhe, so that patterns can be compared. Measured amino acids are categorized into Trophic, Source, and Metabolic (M), based on relative changes with trophic transfer (see main text). Site and amino acid abbreviations are as defined in main text. Overall δ15NAA patterns conform closely to those expected from other heterotrophic organisms, with Trophic AA enriched in 15N vs. Source AA, and Thr strongly depleted in 15N. (TIFF) [file pone.0098087.s002.tiff]

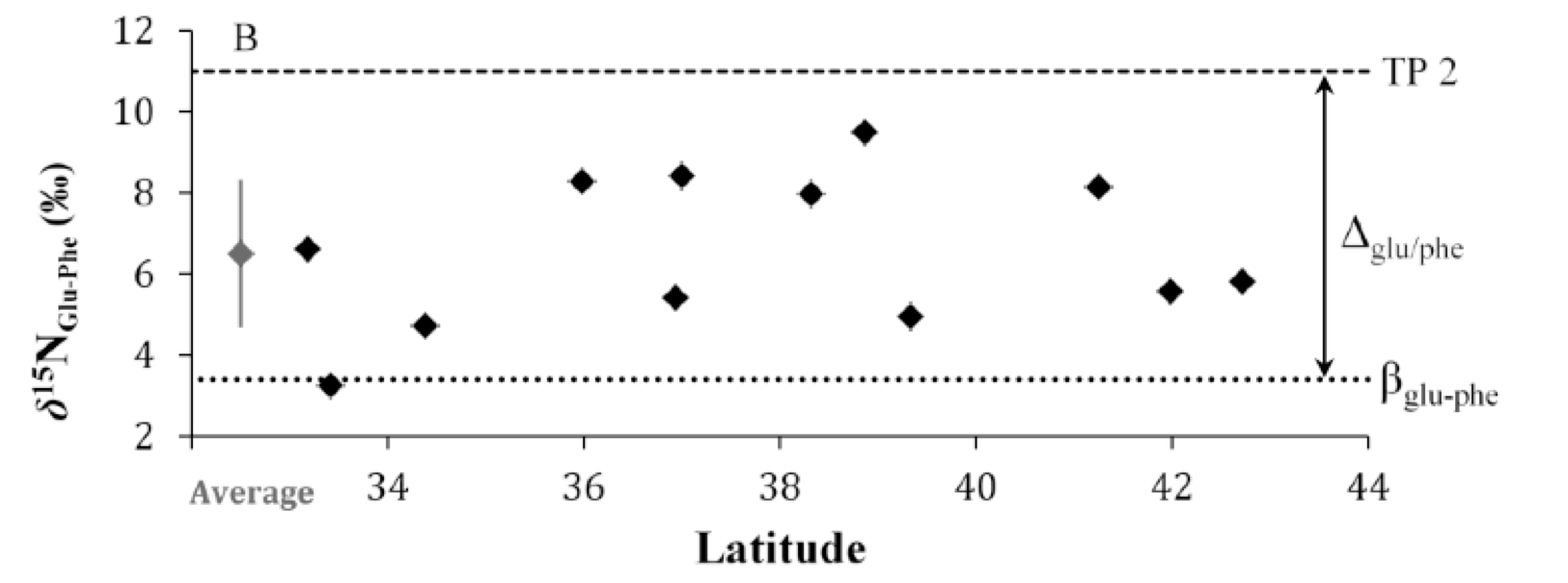

Supplement: Figure S3 — Low CSI-AA based Mussel Trophic Position Results. Relationships between measured δ15NGlu-Phe values vs. expectations for standard CSI-AA TP equations. Measured δ15NGlu-Phe of mussels are plotted vs. latitude (filled diamonds). Shaded bar on average value represents ± 1SD for entire data set. Assumed β values for primary producers are indicated by lower dotted line, (βGlu-Phe, 3.4 per mil). Commonly assumed TEFGlu-Phe for a single trophic transfer for a primary consumer (7.6 per mil ) is represented by upper dashed line. Arrow represents ΔGlu-Phe the theoretical isotopic enrichment from a TP1 to a TP2. (TIFF) [file pone.0098087.s003.tiff]

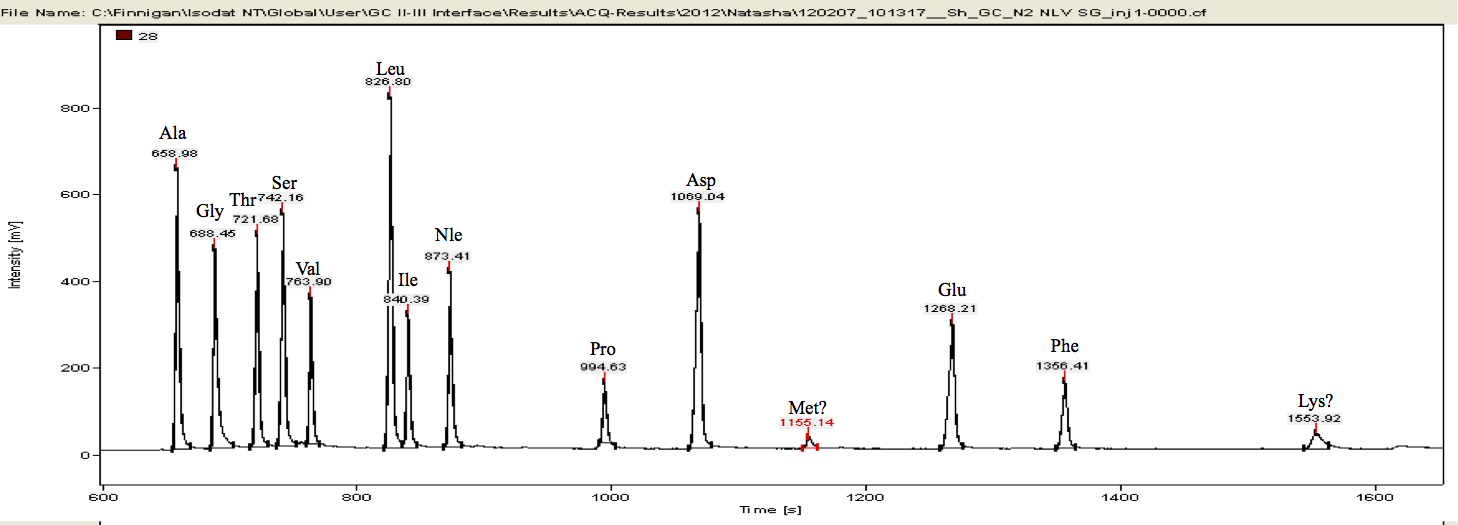

Supplement: Figure S4 — Representative chromatogram of a GC-IRMS analysis of amino acids. Mussel amino acid gas chromatogram. A representative gas chromatogram of derivatized individual amino acids from Mytilus californianus. Abbreviations: Ala, alanine; Gly, Glycine; Thr, threonine; Ser, serine; Val, valine; Leu, leucine; Ile, isoleucine; Nor, Norleucine (internal standard); Pro, proline; Asp, aspartic acid, Met, Methionine; Glu, glutamic acid; Phe, phenylalanine; Lys, Lysine. (TIFF) [file pone.0098087.s004.tiff]
