# Supplementary material for: Compound-Specific δ15N Amino Acid Measurements in Littoral Mussels in the California Upwelling Ecosystem: A New Approach to Generating Baseline δ15N Isoscapes for Coastal Ecosystems
Source: PLoS One. 2014 Jun 2;9(6):e98087. doi: 10.1371/journal.pone.0098087 (PMC4041574; doi:10.1371/journal.pone.0098087)
Supplement: Text S1 — Mussel trophic position discussion. (DOCX) [file pone.0098087.s005.docx]

The underlying reason for the unexpectedly low TP measured in mussels is evident in comparing the offset between Glu-Phe values (δ^15^N _Glu-Phe_; Fig S3). In the commonly used equation for TP (Eq. 1), a 7.6‰ TEF_Glu-Phe_ is assumed, which represents the isotopic enrichment expected for each trophic transfer. However, in primary producers there is also an offset between Glu and Phe δ^15^N values [1,2]. An average value for this offset, commonly termed the ß value, is currently assumed to be 3.4 ‰ [1]. Therefore, using the assumptions of the most common CSIA-AA TP equation, the *minimum* δ^15^N_Glu-Phe_ value for any primary consumer should be approximately 10.9‰ (i.e. the sum of the ß value and TEF_Glu-Phe_; 3.4 + 7.6 = 10.9). However, the substantially lower δ^15^N_Glu-Phe_ we measured in most mussels (Fig. S3; average = 6.5 ± 1.8) is clearly not compatible with these assumptions.

There are two basic interpretations for why the standard TP equation appears to return underestimates for littoral mussels. The first is that the TEF value is overestimated; i.e., that mussels do not fractionate Glu at the 7.6‰ now assumed per trophic transfer. Accumulating literature data in fact now suggests that TEF values may vary, and in some organisms are substantially lower than was assumed based on early work [3,4]. However, lower TEF values have so far been primarily documented for only higher TP animals (e.g., pinnipeds, sharks, birds and turtles [5-7]. Most data reported for lower TP marine animals, including invertebrates, has indicated that canonical TEF values are close to correct (e.g., [1].

An alternate explanation, however, could also be variability in ß values for local primary production. While almost no studies have focused specifically on comparing ß values, recent work has suggested that δ^15^N-AA patterns, including the δ^15^N_Glu-Phe_ offset, can vary systematically between different major groups of primary producers [2], similar to characteristically different patterns in *δ*^13^C-AA between primary producer groups [8,9]. Although a ß_Glu-Phe_ of -3.4‰ appears to be a good average [1,10-12], these is also wide range of variability in some data, and characteristic ß_Glu-Phe_ values for specific algal groups have not yet been closely investigated. For example, if seagrass detritus were a main food source for mussels, this might help to explain the offset we observe. VanderZanden et al. (2013) showed that amino acid biosynthesis of seagrass is more similar to C3 plants, as would be expected, given their close phylogenetic relationship to terrestrial plants. The β_Glu-Phe_ of -3.4‰ for phytoplankton and macroalgae has also been shown to be far different than the value for C_3_ plants (~ +8.4‰; [1]). An elevated β_Glu-Phe_ value would shift the TP mussel closer to the expected 2.0. However, as noted in the main text, substantial seagrass input seem preclude both by CSI-AA fingerprinting, as well as basic ecologic expectations.

Overall, our data does not allow us to evaluate which of these two interpretations is more likely, and we note they are also not mutually exclusive. However, these data do suggest that further investigation of basic assumptions underlying TP equations are needed. In particular, to our knowledge, TEF values for filter feeding mollusks have never been directly investigated.

1. Chikaraishi Y, Ogawa NO, Kashiyama Y, Takano Y, Suga H, et al. (2009) Determination of aquatic food-web structure based on compound-specific nitrogen isotopic composition of amino acids. Limnology and Oceanography: Methods 7: 740–750.

2. McCarthy MD, Lehman J, Kudela R (2013) Compound-specific amino acid Î´15N patterns in marine algae: Tracer potential for cyanobacterial vs. eukaryotic organic nitrogen sources in the ocean. GEOCHIMICA ET COSMOCHIMICA ACTA 103: 104–120. doi:10.1016/j.gca.2012.10.037.

3. Dale JJ, Wallsgrove NJ, Popp BN, Holland KN (2011) Nursery habitat use and foraging ecology of the brown stingray Dasyatis lata determined from stomach contents, bulk and amino acid stable isotopes. Mar Ecol Prog Ser 433: 221–236. doi:10.3354/meps09171.

4. Lorrain A, Graham B, Ménard F, Popp B, Bouillon S, et al. (2009) Nitrogen and carbon isotope values of individual amino acids: a tool to study foraging ecology of penguins in the Southern Ocean. Mar Ecol Prog Ser 391: 293–306. doi:10.3354/meps08215.

5. Germain LR, Koch PL, Harvey J, McCarthy MD (2013) Nitrogen isotope fractionation in amino acids from harbor seals: implications for compound-specific trophic position calculations. Mar Ecol Prog Ser 482: 265–277.

6. Vander Zanden HB, Arthur KE, Bolten AB, Popp BN, Lagueux CJ, et al. (2013) Trophic ecology of a green turtle breeding population. Mar Ecol Prog Ser 476: 237–249. doi:10.3354/meps10185.

7. Madigan DJ, Litvin SY, Popp BN, Carlisle AB, Farwell CJ, et al. (2012) Tissue Turnover Rates and Isotopic Trophic Discrimination Factors in the Endothermic Teleost, Pacific Bluefin Tuna (Thunnus orientalis). PLoS ONE 7: e49220. doi:10.1371/journal.pone.0049220.s002.

8. Larsen T, Wooller MJ, Fogel ML, O'Brien DM (2012) Can amino acid carbon isotope ratios distinguish primary producers in a mangrove ecosystem? Rapid Commun Mass Spectrom 26: 1541–1548. doi:10.1002/rcm.6259.

9. Larsen T, Taylor DL, Leigh MB, O'Brien DM (2009) Stable isotope fingerprinting: a novel method for identifying plant, fungal, or bacterial origins of amino acids. Ecology 90: 3526–3535.

10. McClelland JW, Montoya JP (2002) Trophic relationships and the nitrogen isotopic composition of amino acids in plankton. Ecology 83: 2173–2180.

11. McClelland JW, Holl CM, Montoya JP (2003) Relating low δ15N values of zooplankton to N2-fixation in the tropical North Atlantic: insights provided by stable isotope ratios of amino acids. Deep Sea Research Part I: Oceanographic Research Papers 50: 849–861. doi:10.1016/S0967-0637(03)00073-6.

12. McCarthy MD, Benner R, Lee C, Fogel ML (2007) Amino acid nitrogen isotopic fractionation patterns as indicators of heterotrophy in plankton, particulate, and dissolved organic matter. GEOCHIMICA ET COSMOCHIMICA ACTA 71: 4727–4744. doi:10.1016/j.gca.2007.06.061.
